# Giant Cervicothoracic Madelung Disease Presenting with Airway Compromise: A Case Report from Ethiopia

**DOI:** 10.70352/scrj.cr.25-0802

**Published:** 2026-03-27

**Authors:** Amanuel Mesfin Oljira, Obsa Biratu Negasa, Diriba Gebeyehu Wakesa, Sinbona Ararsa Keneni, Berhanu Nigusse Bikila, Chala Abdo Dammesa, Tesfaye Hurgesa Bayisa, Rabirra Waktola Gonfa, Osman Aman Hamido, Dereje Gurmessa Geleta

**Affiliations:** 1 Department of Surgery, Ambo University College of Medicine and Health Science, Ambo, Ethiopia; 2 Department of Pathology, Ambo University College of Medicine and Health Science, Ambo, Ethiopia; 3 Department of Radiology, Ambo University College of Medicine and Health Science, Ambo, Ethiopia; 4 Department of Anesthesia, Ambo University College of Medicine and Health Science, Ambo, Ethiopia

**Keywords:** multiple symmetric lipomatosis, madelung disease, airway compromise, awake fiberoptic intubation, cervicothoracic mass, staged debulking

## Abstract

**INTRODUCTION:**

To describe a rare (airway-threatening) cervicothoracic presentation of Madelung disease (multiple symmetric lipomatosis) with upper mediastinal extension and to introduce a reproducible peri-operative strategy in a resource-limited setting.

**CASE PRESENTATION:**

We report a case of a 40-year-old Oromo Ethiopian male who presented with progressive cervicothoracic adipose masses resulting in dyspnea and venous congestion. Awake fiberoptic intubation, staged resection, early extubation, and close airway management were the hallmarks of peri-operative management. Contrast-enhanced CT showed non-encapsulated, diffuse adipose infiltration from the neck into the upper mediastinum with effacement of the trachea. Because extensive bilateral cervical dissection posed a high risk of postoperative airway compromise (edema/hematoma) and prolonged anesthesia, a pre-planned two-stage surgical strategy was chosen. Stage 1 anterior debulking removed 3.5 kg of fatty tissue, and Stage 2 posterolateral debulking removed an additional 2.0 kg (total 5.5 kg). There were no major complications. The patient was discharged one week after the second procedure. At 1 month follow-up, there was significant improvement in breathing and daily functioning with no evidence of early recurrence. Clinical and ultrasound follow-up at 3, 6, and 9 months and 1 year showed no recurrence or complications.

**CONCLUSIONS:**

Giant cervicothoracic Madelung disease can lead to critical airway compromise. Planned awake fiberoptic intubation and staged debulking resulted in safe care and good outcomes. Reporting detailed airway and surgical strategies for this uncommon presentation may be helpful to teams working in resource-constrained settings. Long-term surveillance remains essential because postoperative recurrence has been reported.

## Abbreviations

ASA
: American Society of Anesthesiologists
EBL
: estimated blood loss
ETT
: endotracheal tube
HDU
: high-dependency unit
IHC
: immunohistochemistry
IV
: intravenous
PRBC
: packed red blood cells

## INTRODUCTION

Madelung disease (multiple symmetric lipomatosis; Launois–Bensaude syndrome) is a rare form of lipodystrophy characterized by diffuse, symmetric, non-encapsulated fatty deposits involving mainly the neck, shoulders, and upper trunk. It predominantly affects middle-aged men, and chronic alcohol consumption is frequently associated.^1–3)^

CT and MRI play a central role in diagnosis and pre-operative planning by delineating the extent, symmetry, and relationship to aerodigestive structures.^4,5)^ Cervicothoracic involvement can cause airway, esophageal, or venous compression requiring multispecialty management and sometimes urgent airway intervention.^6)^

We present a case of a giant cervicothoracic presentation with airway compromise successfully managed with awake fiberoptic intubation and staged surgical debulking, focusing on peri-operative steps practical in resource-limited settings.

## CASE PRESENTATION

**Fig. 1:**
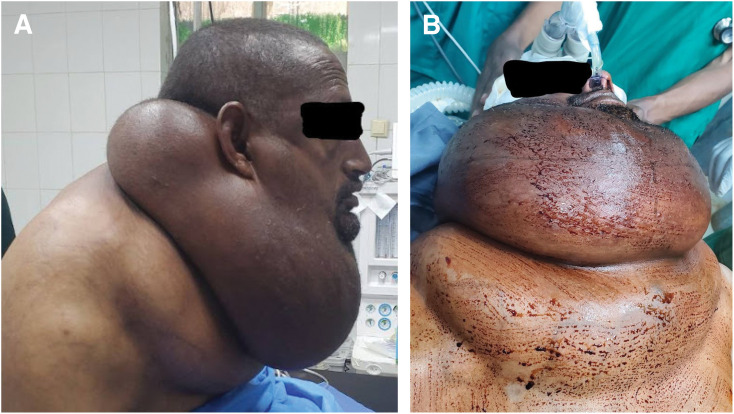
Giant circumferential cervicothoracic mass. (**A**) Lateral view. (**B**) Supine anteroposterior view with a nasotracheal tube in place.

A 40-year-old Ethiopian male presented with a 10-year history of progressive neck mass extending anteriorly and posteriorly to the chest wall. Associated symptoms included dyspnea, functional limitation, and cosmetic deformity. The patient has admitted to habitual intake of alcohol over the past 20 years. Examination documented a giant circumferential cervicothoracic mass with a maximum neck circumference of 105 cm and venous congestion suggestive of superior mediastinal extension (**Fig. 1A**; **Table 1**).

**Table 1:** Timeline of symptoms and management

Timepoint	Symptoms & signs	Key clinical events	Management	Outcome/notes
20 years before admission	Regular alcohol consumption; no MD symptoms			Risk factor acknowledged; later counseled to stop alcohol
10 years before admission	Gradual neck enlargement; no dyspnea initially			Slow progression
3–2 years before admission	Increasing bulk; cosmetic concerns; exertional breathlessness			Functional impact emerges
6–3 months before admission	Worsening dyspnea (supine), reduced neck mobility, venous fullness	Preoperative surgical and anesthesia evaluation	Preoperative planning for anticipated difficult airway and staged surgery	Supine intolerance noted
Admission (D–1 to D0)	Marked cervicothoracic enlargement; positional dyspnea	CT confirmed diffuse non-encapsulated fat with upper mediastinal extension and tracheal effacement; ASA III	Planned awake fiberoptic nasotracheal intubation and staged debulking	Anticipated difficult airway
D0 (Stage 1 surgery)	Dyspnea and compressive symptoms	Awake fiberoptic nasotracheal intubation; anterior debulking	Excision ~3.5 kg; EBL 500 mL; 1 unit PRBC transfused intraoperatively; Penrose drain	Mild transient stridor treated with nebulization; ICU/HDU monitoring
D+1 to D+2	Improving breathing	Postoperative monitoring	Penrose drain output ~80 mL hemorrhagic over 2 days	Drain removed on POD 2
D+7 (Stage 2 surgery)	Residual posterolateral/posterior bulk	Second-stage debulking	Additional ~2.0 kg excised; EBL 300 mL; Penrose drain	Uneventful recovery
Post-op day 1–2 (Stage 2)	Stable	Postoperative monitoring	Penrose drain output ~50 mL over 2 days	Drain removed on POD 2
Discharge	Improved breathing and function	Discharged one week after Stage 2	Counseling on recurrence and strict alcohol cessation	Stable
1 month	Marked functional and cosmetic improvement	Outpatient review	Continued counseling	No early recurrence
3 months	No symptoms	Clinical + US follow-up	Surveillance	No recurrence/complications
6 months	No symptoms	Clinical + US follow-up	Surveillance	No recurrence/complications
9 months	No symptoms	Clinical + US follow-up	Surveillance	No recurrence/complications
1 year	No symptoms	Clinical + US follow-up	Surveillance	No recurrence/complications; patient remained abstinent from alcohol

**Fig. 2:**
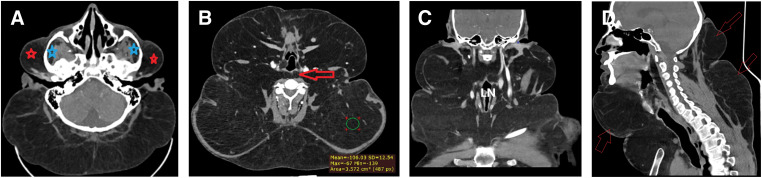
Neck CT scan. (**A**) Axial view at the level of the posterior fossa showing extensive non-encapsulated subcutaneous fat deposition extending bilaterally to parotid (red asterisks) and masticator spaces (blue asterisks). (**B**) Axial view at the level of the larynx showing fat deposition deep to sternocleidomastoid muscles bilaterally and in the prevertebral space (red arrow). (**C**) Post-contrast coronal view showing extensive non-encapsulated subcutaneous fat deposition. (**D**) Sagittal view showing anterior and posterior neck involvement with posterior “buffalo hump” appearance (red arrows).

Contrast-enhanced CT of the neck and upper chest showed large, non-encapsulated fat tissue filling the cervicothoracic region and spreading to the retrosternal/upper mediastinal spaces with tracheal effacement (**Fig. 2**). Imaging findings were consistent with Madelung disease and guided surgical planning.

Given the anticipated difficult airway due to the cervicothoracic mass effect, the patient was classified as ASA III. After counseling and written informed consent, awake fiberoptic intubation was planned.

The patient was placed sitting due to dyspnea in the supine position. Standard monitoring and supplemental oxygen were provided. Airway topicalization included nebulized lidocaine (4 mL of 2%), gargling of viscous lidocaine, nasal mucosa desensitization using lidocaine–adrenaline spray, and 0.2 mg of glycopyrrolate IV to reduce secretions. Sedation consisted of 1 mg of midazolam IV. A flexible fiberoptic bronchoscope guided placement of a 6.5-mm ETT via the nasal cavity under direct visualization (**Fig. 1B**). After securing the airway, the patient was repositioned supine with a shoulder roll and neck extension for surgery. General anesthesia was initiated with propofol (2 mg/kg) and fentanyl (2 μg/kg), maintained with isoflurane and IV analgesics.

### Surgical management (two-stage strategy)

The procedure was pre-planned as two stages to reduce postoperative airway risk (edema/hematoma) after extensive bilateral cervical dissection, avoid prolonged anesthesia, improve hemostatic control, and address multi-compartment disease systematically. A 1-week interval was chosen to permit edema resolution, confirm airway stability, and allow recovery before the second stage.

**Stage 1 (anterior debulking):** A transverse cervical collar (apron-type) incision was used. Dissection proceeded in the subplatysmal plane and along cervical fascial planes with early identification and protection of the carotid sheath; the trachea and esophagus were visualized and preserved using careful blunt and sharp dissection. Hemostasis used monopolar cautery (superficial), bipolar cautery (near vital structures), and suture ligatures for larger vessels, with frequent hemostatic checks; diluted adrenaline-containing local anesthetic infiltration was used to reduce bleeding.

**Fig. 3:**
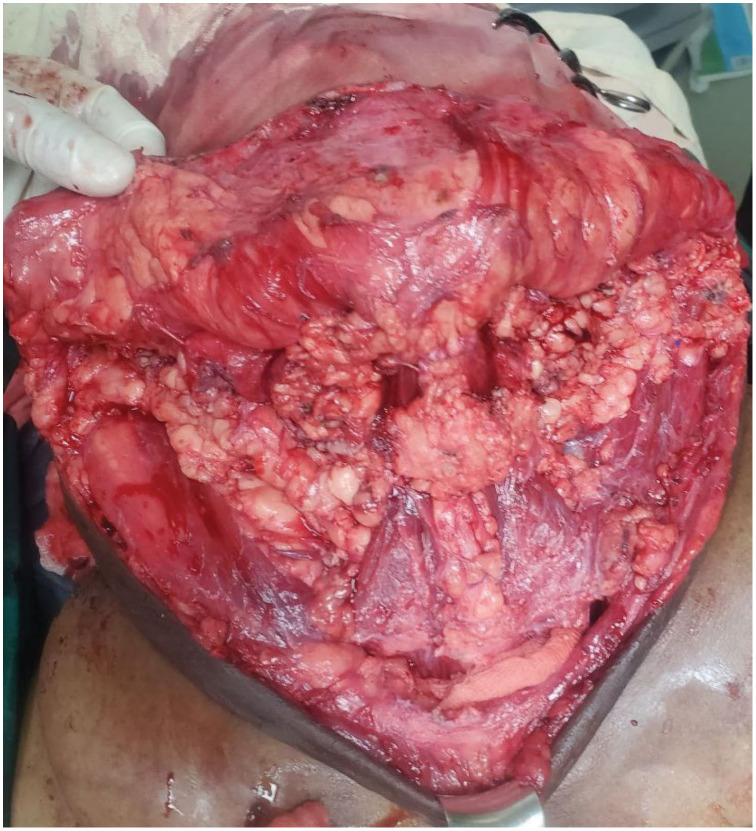
Intraoperative picture showing adipose tissue dissection in the anterior neck with preservation of vital structures.

Meticulous debulking removed approximately 3.5 kg of lipomatous tissue invading the strap muscles and surrounding the carotid sheath, trachea, and esophagus (**Fig. 3**). EBL was 500 mL, and 1 unit of PRBC was transfused intraoperatively during stage 1. A Penrose drain was placed; drain output was hemorrhagic, totaling ~80 mL over 2 days, and it was removed on POD 2. Extubation was performed after full awakening and reversal; 6 mg of dexamethasone IV was administered prior to extubation, and airway equipment was prepared for reintubation. The patient developed a mild transient stridor, was treated with nebulization (dexamethasone 6 mg + adrenaline 1 mg), and was monitored in ICU/HDU, remaining stable.

**Stage 2 (posterolateral/posterior debulking; 1 week later):** A further 2.0 kg of adipose tissue was removed from the lateral and posterior compartments of the neck. EBL was 300 mL. A Penrose drain was used again; output totaled ~50 mL over 2 days and was removed on POD 2. Recovery was smooth, and the patient was discharged 1 week after the second procedure.

### Pathology

**Fig. 4:**
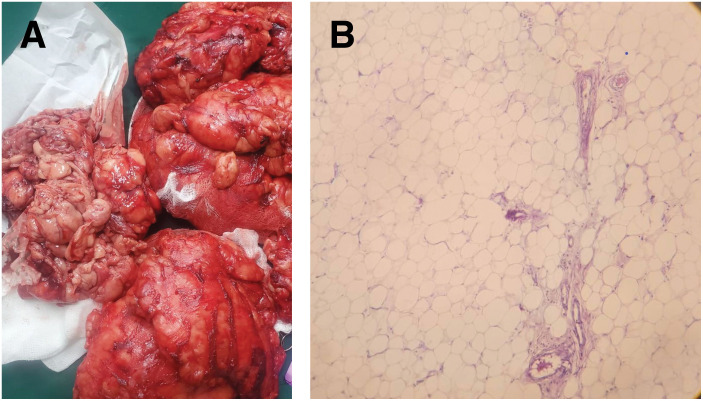
Histology of the resected adipose tissue. (**A**) Gross specimen showing non-encapsulated adipose tissue. (**B**) Microscopy showing lobules of mature adipose tissue composed of benign mature adipocytes.

The final biopsy showed lobules of mature adipose tissue with benign mature adipocytes and areas of fibrous tissue with a well-delineated margin but without a capsule. No nuclear atypia, lipoblasts, or necrosis were identified, and IHC was not available (**Fig. 4**).

### Follow-up

**Fig. 5:**
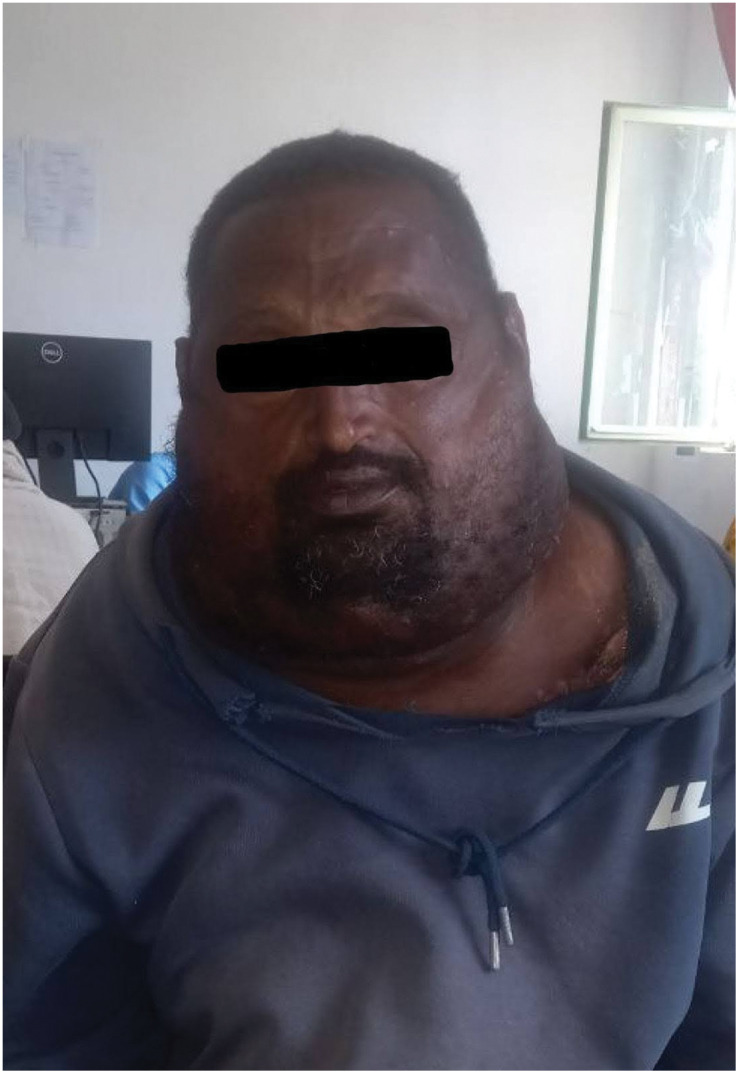
Postoperative picture showing reduced neck mass.

At 1 month, the patient reported significant improvement in breathing and ADL with marked cosmetic improvement and no early recurrence (**Table 1**; **Fig. 5**). He was explicitly counseled about recurrence risk and counseled to stop alcohol; he stopped alcohol consumption postoperatively. Follow-up at 3, 6, and 9 months and 1 year (clinical and US-based) demonstrated no recurrence or complications.

## DISCUSSION

Madelung disease involves symmetric, non-encapsulated adipose deposition classically in the cervicothoracic distribution (Type I/Enzi pattern). Long-term alcohol use is frequently associated, although causality is uncertain.^1–3)^ A systematic review highlights hypotheses including catecholamine-related lipolytic pathway defects and mitochondrial dysfunction and recommends alcohol cessation as part of comprehensive management because alcohol may aggravate progression.^1)^ In our patient, alcohol cessation was achieved postoperatively, and recurrence counseling was explicitly provided.

CT/MRI defines the extent and relationship to airway/vascular structures and helps distinguish Madelung disease from liposarcoma, Dercum disease, familial multiple lipomatosis, and other lipodystrophies.^4,5)^

Surgery (open debulking or liposuction) remains the mainstay when compression or disfigurement is significant. Recurrence after surgery is reported; a systematic review found an overall recurrence of approximately 18%.^1)^ A recent case series advocates staged excision and careful intraoperative management to optimize outcomes and reduce bleeding/complications.^7)^ In our patient, the two-stage plan was pre-planned to mitigate airway/hematoma risk and limit operative stress per session, with structured follow-up to 1 year showing no recurrence.

For airway-threatening cervicothoracic disease, awake fiberoptic intubation preserves spontaneous ventilation and visualization, reducing induction risk. ASA guidance emphasizes preoxygenation, continuous oxygen delivery, and extubation planning with readiness for reintubation.^8)^ In resource-limited settings without advanced rescue devices, explicit backup planning and multidisciplinary readiness are critical. Feasibility protocols and training-based approaches support consistent performance of awake fiberoptic techniques.^9,10)^

**Pathologic differentiation:** The benign histology (mature adipocytes without atypia, lipoblasts, or necrosis) supports Madelung disease rather than well-differentiated liposarcoma; in settings where available, IHC (e.g., MDM2/CDK4) may help exclude malignancy when morphology is equivocal. IHC was not available in our setting.

## CONCLUSION

Giant cervicothoracic Madelung disease may result in life-threatening airway compromise. In this patient, an approach of awake fiberoptic intubation with staged debulking achieved excellent early functional improvement and sustained benefit. Detailed reporting of airway and operative steps may help teams in resource-constrained environments manage rare high-risk cervicothoracic lipomatosis. Long-term surveillance (≥12 months) is recommended given documented recurrence risk, and our patient remained recurrence-free at 1 year with clinical and US follow-up.

### Patient perspective

“I am happy with the cosmetic result and the breathing and daily activities have improved markedly.”

## DECLARATIONS

### Data availability

The data that support the findings of this study are available from the corresponding author upon reasonable request.
